# Ganges: special at its origin

**DOI:** 10.1186/s40709-016-0055-6

**Published:** 2016-07-15

**Authors:** Krishna Khairnar

**Affiliations:** Environmental Virology Cell, Council of Scientific and Industrial Research-National Environmental Engineering Research Institute, Room-112, Nehru Marg, Nagpur, Maharashtra 440020 India

River Ganges in India, for centuries, has been revered for its “self-cleansing and special healing properties”. More than 450 million people depend on the waters of Ganges for many aspects of their life. In 1896, one of the first published works on Ganges water by Ernst Hankin, a British bacteriologist demonstrated antibacterial property of Ganges water against *Vibrio cholera* [1]. Further work by French microbiologist D’Herelles in the beginning of the twentieth century established that the antibacterial property of Ganges water to be due to a factor, later named “bacteriophage” [2]. The above said studies were conducted during the period of Crown rule in India and a significant number of founding studies on Ganges were carried out mainly by British and French microbiologists. Such studies on Ganges led to the introduction of bacteriophages to the world. Bacteriophages are the prokaryotic viruses that solely infect and/or destroy the bacteria. Bacteriophages were associated with the special property of river Ganges [1, 2].

**Fig. 1:**
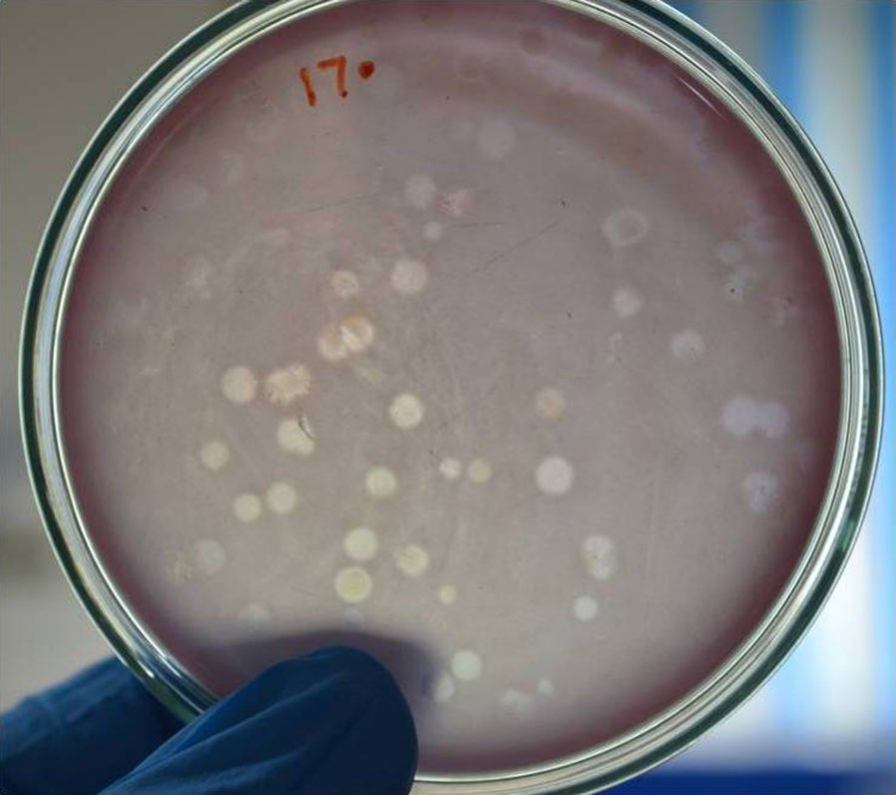
Representative plate of phages isolated against bacteria—*Escherichia* spp. at Gomukh’s melting permafrost. Plaque assay—clear plaques due to bacteriophage mediated bacterial cell lysis are visible

Interestingly, our study for the first time has shown the presence of bacteriophages against putrefying and pathogenic bacteria in the waters of Ganges even at its origin (Fig. 1). The origin of river Ganges is known as Gomukh. Geological studies have proven that the Himalayas had emerged at a site where the Tethys Ocean once existed, as a result of collision between the Indian and Eurasian tectonic plates. Hence, Himalayas’ marine origin is known and it has been intriguing to find marine fossils high in the Himalayas [3].

Notably, the physical property of water at the Gomukh is also unique, as observed during the sampling, loads of sediment gushes out in force along with water at this origin site which is due to the melting permafrost. Typically the Himalayan permafrost [4] melts and forms the origin of Ganges. We believe the bacteriophages trapped at a much earlier time scale in the Himalayan permafrost as abiotic particles are being released gradually with the melting permafrost, thereby making a seed source of bacteriophage at Gomukh. To the best of our knowledge this is the first ground breaking finding of its kind.

A recent publication shows that 30,000 years old frozen giant virus was discovered deep in the Siberian Permafrost, was revived and still infectious [5]. Interestingly, another group has isolated a temperate bacteriophage from Antarctic Dry Valley environment against *Psychrobacter*—an extremophile and studied its genomic aspects [6]. The above stated findings literally encourage and prompt for further groundbreaking possibilities with exploring bacteriophages and its revival in the frozen Himalayan permafrost.
